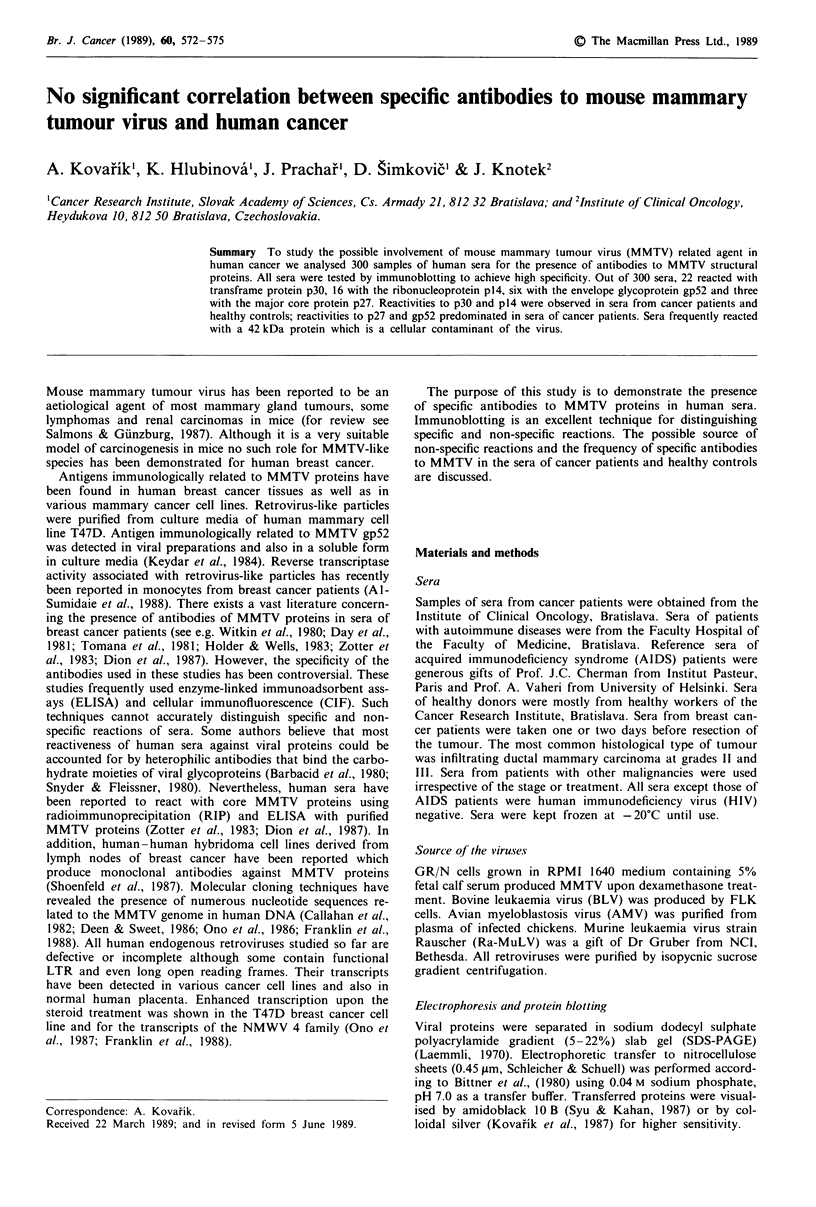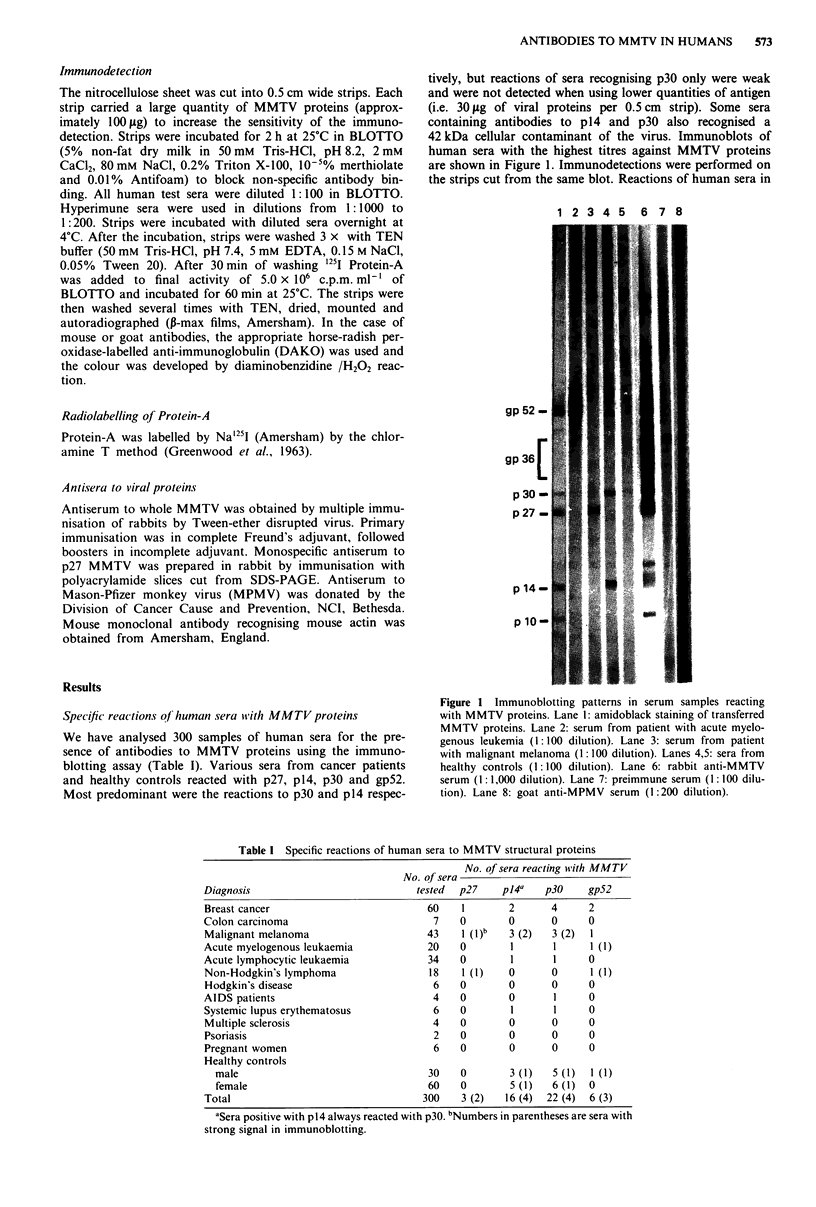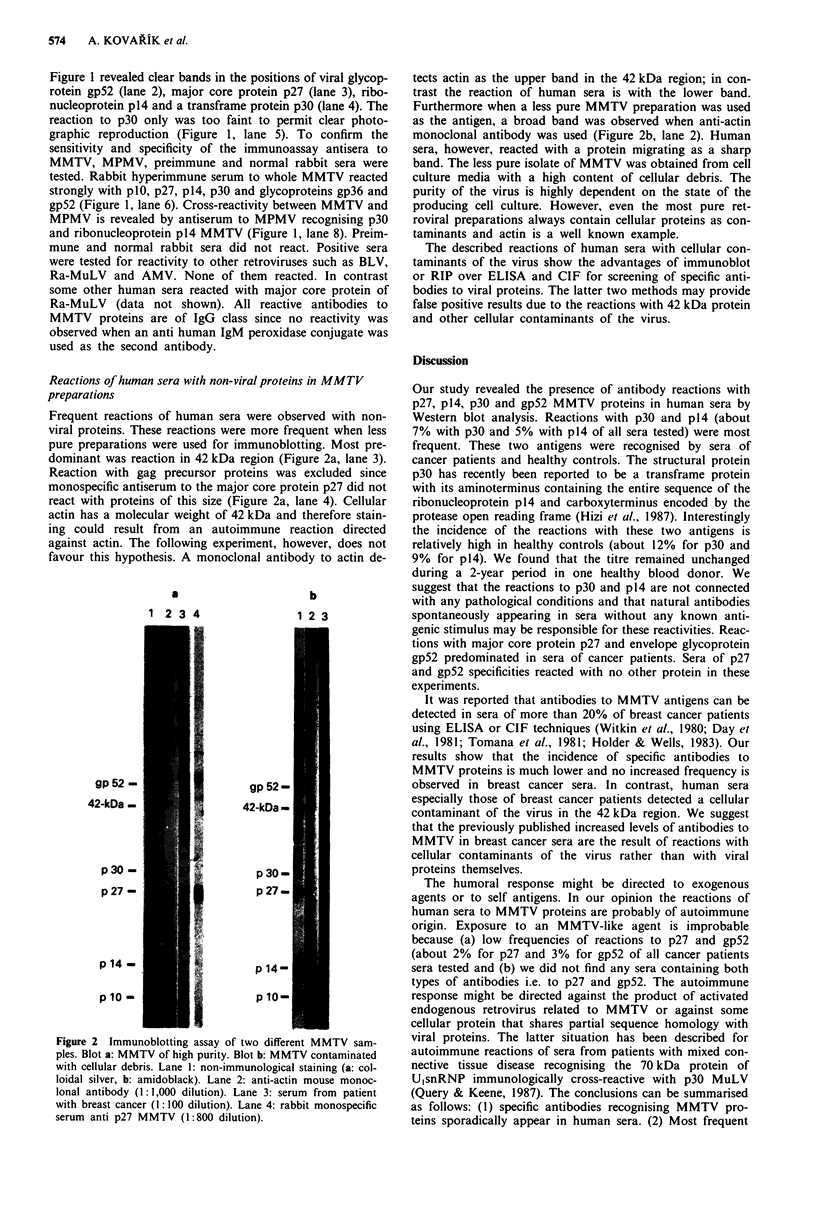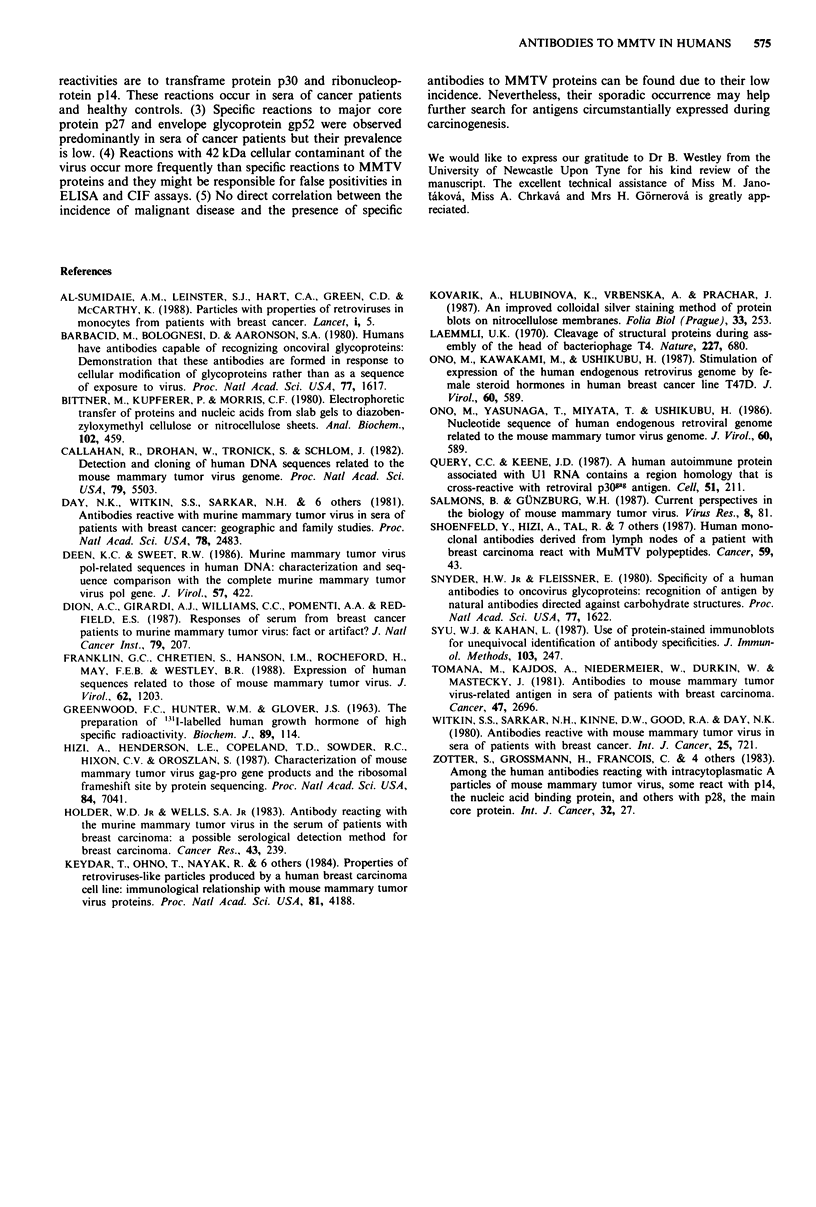# No significant correlation between specific antibodies to mouse mammary tumour virus and human cancer.

**DOI:** 10.1038/bjc.1989.315

**Published:** 1989-10

**Authors:** A. KovarÃ­k, K. HlubinovÃ¡, J. Prachar, D. Simkovic, J. Knotek

**Affiliations:** Cancer Research Institute, Slovak Academy of Sciences, Bratislava, Czechoslovakia.

## Abstract

**Images:**


Br. J. Cancer (1989), 60, 572-575 © The Macmillan Press Ltd., 1989

### No significant correlation between specific antibodies to mouse mammary tumour virus and human cancer

A. Kovařík[1], K. Hlubinová[1], J. Prachař[1], D. Šimkovič[1] & J. Knotek[2]

[1]*Cancer Research Institute, Slovak Academy of Sciences, Cs. Armady 21, 812 32 Bratislava; and* [2]*Institute of Clinical Oncology, Heydukova 10, 812 50 Bratislava, Czechoslovakia.*

**Summary** To study the possible involvement of mouse mammary tumour virus (MMTV) related agent in human cancer we analysed 300 samples of human sera for the presence of antibodies to MMTV structural proteins. All sera were tested by immunoblotting to achieve high specificity. Out of 300 sera, 22 reacted with transframe protein p30, 16 with the ribonucleoprotein p14, six with the envelope glycoprotein gp52 and three with the major core protein p27. Reactivities to p30 and p14 were observed in sera from cancer patients and healthy controls; reactivities to p27 and gp52 predominated in sera of cancer patients. Sera frequently reacted with a 42 kDa protein which is a cellular contaminant of the virus.

Mouse mammary tumour virus has been reported to be an aetiological agent of most mammary gland tumours, some lymphomas and renal carcinomas in mice (for review see Salmons & Günzburg, 1987). Although it is a very suitable model of carcinogenesis in mice no such role for MMTV-like species has been demonstrated for human breast cancer.

Antigens immunologically related to MMTV proteins have been found in human breast cancer tissues as well as in various mammary cancer cell lines. Retrovirus-like particles were purified from culture media of human mammary cell line T47D. Antigen immunologically related to MMTV gp52 was detected in viral preparations and also in a soluble form in culture media (Keydar *et al.*, 1984). Reverse transcriptase activity associated with retrovirus-like particles has recently been reported in monocytes from breast cancer patients (Al-Sumidaie *et al.*, 1988). There exists a vast literature concerning the presence of antibodies of MMTV proteins in sera of breast cancer patients (see e.g. Witkin *et al.*, 1980; Day *et al.*, 1981; Tomana *et al.*, 1981; Holder & Wells, 1983; Zotter *et al.*, 1983; Dion *et al.*, 1987). However, the specificity of the antibodies used in these studies has been controversial. These studies frequently used enzyme-linked immunoadsorbent assays (ELISA) and cellular immunofluorescence (CIF). Such techniques cannot accurately distinguish specific and non-specific reactions of sera. Some authors believe that most reactiveness of human sera against viral proteins could be accounted for by heterophilic antibodies that bind the carbohydrate moieties of viral glycoproteins (Barbacid *et al.*, 1980; Snyder & Fleissner, 1980). Nevertheless, human sera have been reported to react with core MMTV proteins using radioimmunoprecipitation (RIP) and ELISA with purified MMTV proteins (Zotter *et al.*, 1983; Dion *et al.*, 1987). In addition, human-human hybridoma cell lines derived from lymph nodes of breast cancer have been reported which produce monoclonal antibodies against MMTV proteins (Shoenfeld *et al.*, 1987). Molecular cloning techniques have revealed the presence of numerous nucleotide sequences related to the MMTV genome in human DNA (Callahan *et al.*, 1982; Deen & Sweet, 1986; Ono *et al.*, 1986; Franklin *et al.*, 1988). All human endogenous retroviruses studied so far are defective or incomplete although some contain functional LTR and even long open reading frames. Their transcripts have been detected in various cancer cell lines and also in normal human placenta. Enhanced transcription upon the steroid treatment was shown in the T47D breast cancer cell line and for the transcripts of the NMWV 4 family (Ono *et al.*, 1987; Franklin *et al.*, 1988).

Correspondence: A. Kovařík.
Received 22 March 1989; and in revised form 5 June 1989.

The purpose of this study is to demonstrate the presence of specific antibodies to MMTV proteins in human sera. Immunoblotting is an excellent technique for distinguishing specific and non-specific reactions. The possible source of non-specific reactions and the frequency of specific antibodies to MMTV in the sera of cancer patients and healthy controls are discussed.

### Materials and methods

### *Sera*

Samples of sera from cancer patients were obtained from the Institute of Clinical Oncology, Bratislava. Sera of patients with autoimmune diseases were from the Faculty Hospital of the Faculty of Medicine, Bratislava. Reference sera of acquired immunodeficiency syndrome (AIDS) patients were generous gifts of Prof. J.C. Cherman from Institut Pasteur, Paris and Prof. A. Vaheri from University of Helsinki. Sera of healthy donors were mostly from healthy workers of the Cancer Research Institute, Bratislava. Sera from breast cancer patients were taken one or two days before resection of the tumour. The most common histological type of tumour was infiltrating ductal mammary carcinoma at grades II and III. Sera from patients with other malignancies were used irrespective of the stage or treatment. All sera except those of AIDS patients were human immunodeficiency virus (HIV) negative. Sera were kept frozen at $-20$°C until use.

### *Source of the viruses*

GR/N cells grown in RPMI 1640 medium containing 5% fetal calf serum produced MMTV upon dexamethasone treatment. Bovine leukaemia virus (BLV) was produced by FLK cells. Avian myeloblastosis virus (AMV) was purified from plasma of infected chickens. Murine leukaemia virus strain Rauscher (Ra-MuLV) was a gift of Dr Gruber from NCI, Bethesda. All retroviruses were purified by isopycnic sucrose gradient centrifugation.

### *Electrophoresis and protein blotting*

Viral proteins were separated in sodium dodecyl sulphate polyacrylamide gradient (5-22%) slab gel (SDS-PAGE) (Laemmli, 1970). Electrophoretic transfer to nitrocellulose sheets (0.45 µm, Schleicher & Schuell) was performed according to Bittner *et al.*, (1980) using 0.04 M sodium phosphate, pH 7.0 as a transfer buffer. Transferred proteins were visualised by amidoblack 10 B (Syu & Kahan, 1987) or by colloidal silver (Kovařík *et al.*, 1987) for higher sensitivity.

*Immunodetection*

The nitrocellulose sheet was cut into 0.5 cm wide strips. Each strip carried a large quantity of MMTV proteins (approximately 100 μg) to increase the sensitivity of the immunodetection. Strips were incubated for 2 h at 25°C in BLOTTO (5% non-fat dry milk in 50 mM Tris-HCl, pH 8.2, 2 mM $CaCl_2$, 80 mM NaCl, 0.2% Triton X-100, $10^{-5}$% merthiolate and 0.01% Antifoam) to block non-specific antibody binding. All human test sera were diluted 1:100 in BLOTTO. Hyperimune sera were used in dilutions from 1:1000 to 1:200. Strips were incubated with diluted sera overnight at 4°C. After the incubation, strips were washed 3 × with TEN buffer (50 mM Tris-HCl, pH 7.4, 5 mM EDTA, 0.15 M NaCl, 0.05% Tween 20). After 30 min of washing $^{125}I$ Protein-A was added to final activity of $5.0 \times 10^6$ c.p.m. $ml^{-1}$ of BLOTTO and incubated for 60 min at 25°C. The strips were then washed several times with TEN, dried, mounted and autoradiographed (β-max films, Amersham). In the case of mouse or goat antibodies, the appropriate horse-radish peroxidase-labelled anti-immunoglobulin (DAKO) was used and the colour was developed by diaminobenzidine /$H_2O_2$ reaction.

*Radiolabelling of Protein-A*

Protein-A was labelled by $Na^{125}I$ (Amersham) by the chloramine T method (Greenwood *et al.*, 1963).

*Antisera to viral proteins*

Antiserum to whole MMTV was obtained by multiple immunisation of rabbits by Tween-ether disrupted virus. Primary immunisation was in complete Freund's adjuvant, followed boosters in incomplete adjuvant. Monospecific antiserum to p27 MMTV was prepared in rabbit by immunisation with polyacrylamide slices cut from SDS-PAGE. Antiserum to Mason-Pfizer monkey virus (MPMV) was donated by the Division of Cancer Cause and Prevention, NCI, Bethesda. Mouse monoclonal antibody recognising mouse actin was obtained from Amersham, England.

### Results

*Specific reactions of human sera with MMTV proteins*

We have analysed 300 samples of human sera for the presence of antibodies to MMTV proteins using the immunoblotting assay (Table I). Various sera from cancer patients and healthy controls reacted with p27, p14, p30 and gp52. Most predominant were the reactions to p30 and p14 respectively, but reactions of sera recognising p30 only were weak and were not detected when using lower quantities of antigen (i.e. 30 μg of viral proteins per 0.5 cm strip). Some sera containing antibodies to p14 and p30 also recognised a 42 kDa cellular contaminant of the virus. Immunoblots of human sera with the highest titres against MMTV proteins are shown in Figure 1. Immunodetections were performed on the strips cut from the same blot. Reactions of human sera in

**Figure 1** Immunoblotting patterns in serum samples reacting with MMTV proteins. Lane 1: amidoblack staining of transferred MMTV proteins. Lane 2: serum from patient with acute myelogenous leukemia (1:100 dilution). Lane 3: serum from patient with malignant melanoma (1:100 dilution). Lanes 4,5: sera from healthy controls (1:100 dilution). Lane 6: rabbit anti-MMTV serum (1:1,000 dilution). Lane 7: preimmune serum (1:100 dilution). Lane 8: goat anti-MPMV serum (1:200 dilution).

**Table I** Specific reactions of human sera to MMTV structural proteins

| Diagnosis | No. of sera tested | No. of sera reacting with MMTV p27 | p14[a] | p30 | gp52 |
|---|---|---|---|---|---|
| Breast cancer | 60 | 1 | 2 | 4 | 2 |
| Colon carcinoma | 7 | 0 | 0 | 0 | 0 |
| Malignant melanoma | 43 | 1 (1)[b] | 3 (2) | 3 (2) | 1 |
| Acute myelogenous leukaemia | 20 | 0 | 1 | 1 | 1 (1) |
| Acute lymphocytic leukaemia | 34 | 0 | 1 | 1 | 0 |
| Non-Hodgkin's lymphoma | 18 | 1 (1) | 0 | 0 | 1 (1) |
| Hodgkin's disease | 6 | 0 | 0 | 0 | 0 |
| AIDS patients | 4 | 0 | 0 | 1 | 0 |
| Systemic lupus erythematosus | 6 | 0 | 1 | 1 | 0 |
| Multiple sclerosis | 4 | 0 | 0 | 0 | 0 |
| Psoriasis | 2 | 0 | 0 | 0 | 0 |
| Pregnant women | 6 | 0 | 0 | 0 | 0 |
| Healthy controls | | | | | |
| male | 30 | 0 | 3 (1) | 5 (1) | 1 (1) |
| female | 60 | 0 | 5 (1) | 6 (1) | 0 |
| Total | 300 | 3 (2) | 16 (4) | 22 (4) | 6 (3) |

[a]Sera positive with p14 always reacted with p30. [b]Numbers in parentheses are sera with strong signal in immunoblotting.

Figure 1 revealed clear bands in the positions of viral glycoprotein gp52 (lane 2), major core protein p27 (lane 3), ribonucleoprotein p14 and a transframe protein p30 (lane 4). The reaction to p30 only was too faint to permit clear photographic reproduction (Figure 1, lane 5). To confirm the sensitivity and specificity of the immunoassay antisera to MMTV, MPMV, preimmune and normal rabbit sera were tested. Rabbit hyperimmune serum to whole MMTV reacted strongly with p10, p27, p14, p30 and glycoproteins gp36 and gp52 (Figure 1, lane 6). Cross-reactivity between MMTV and MPMV is revealed by antiserum to MPMV recognising p30 and ribonucleoprotein p14 MMTV (Figure 1, lane 8). Preimmune and normal rabbit sera did not react. Positive sera were tested for reactivity to other retroviruses such as BLV, Ra-MuLV and AMV. None of them reacted. In contrast some other human sera reacted with major core protein of Ra-MuLV (data not shown). All reactive antibodies to MMTV proteins are of IgG class since no reactivity was observed when an anti human IgM peroxidase conjugate was used as the second antibody.

*Reactions of human sera with non-viral proteins in MMTV preparations*

Frequent reactions of human sera were observed with non-viral proteins. These reactions were more frequent when less pure preparations were used for immunoblotting. Most predominant was reaction in 42 kDa region (Figure 2a, lane 3). Reaction with gag precursor proteins was excluded since monospecific antiserum to the major core protein p27 did not react with proteins of this size (Figure 2a, lane 4). Cellular actin has a molecular weight of 42 kDa and therefore staining could result from an autoimmune reaction directed against actin. The following experiment, however, does not favour this hypothesis. A monoclonal antibody to actin detects actin as the upper band in the 42 kDa region; in contrast the reaction of human sera is with the lower band. Furthermore when a less pure MMTV preparation was used as the antigen, a broad band was observed when anti-actin monoclonal antibody was used (Figure 2b, lane 2). Human sera, however, reacted with a protein migrating as a sharp band. The less pure isolate of MMTV was obtained from cell culture media with a high content of cellular debris. The purity of the virus is highly dependent on the state of the producing cell culture. However, even the most pure retroviral preparations always contain cellular proteins as contaminants and actin is a well known example.

The described reactions of human sera with cellular contaminants of the virus show the advantages of immunoblot or RIP over ELISA and CIF for screening of specific antibodies to viral proteins. The latter two methods may provide false positive results due to the reactions with 42 kDa protein and other cellular contaminants of the virus.

**Figure 2** Immunoblotting assay of two different MMTV samples. Blot **a**: MMTV of high purity. Blot **b**: MMTV contaminated with cellular debris. Lane 1: non-immunological staining (**a**: colloidal silver, **b**: amidoblack). Lane 2: anti-actin mouse monoclonal antibody (1:1,000 dilution). Lane 3: serum from patient with breast cancer (1:100 dilution). Lane 4: rabbit monospecific serum anti p27 MMTV (1:800 dilution).

### Discussion

Our study revealed the presence of antibody reactions with p27, p14, p30 and gp52 MMTV proteins in human sera by Western blot analysis. Reactions with p30 and p14 (about 7% with p30 and 5% with p14 of all sera tested) were most frequent. These two antigens were recognised by sera of cancer patients and healthy controls. The structural protein p30 has recently been reported to be a transframe protein with its aminoterminus containing the entire sequence of the ribonucleoprotein p14 and carboxyterminus encoded by the protease open reading frame (Hizi *et al*., 1987). Interestingly the incidence of the reactions with these two antigens is relatively high in healthy controls (about 12% for p30 and 9% for p14). We found that the titre remained unchanged during a 2-year period in one healthy blood donor. We suggest that the reactions to p30 and p14 are not connected with any pathological conditions and that natural antibodies spontaneously appearing in sera without any known antigenic stimulus may be responsible for these reactivities. Reactions with major core protein p27 and envelope glycoprotein gp52 predominated in sera of cancer patients. Sera of p27 and gp52 specificities reacted with no other protein in these experiments.

It was reported that antibodies to MMTV antigens can be detected in sera of more than 20% of breast cancer patients using ELISA or CIF techniques (Witkin *et al*., 1980; Day *et al*., 1981; Tomana *et al*., 1981; Holder & Wells, 1983). Our results show that the incidence of specific antibodies to MMTV proteins is much lower and no increased frequency is observed in breast cancer sera. In contrast, human sera especially those of breast cancer patients detected a cellular contaminant of the virus in the 42 kDa region. We suggest that the previously published increased levels of antibodies to MMTV in breast cancer sera are the result of reactions with cellular contaminants of the virus rather than with viral proteins themselves.

The humoral response might be directed to exogenous agents or to self antigens. In our opinion the reactions of human sera to MMTV proteins are probably of autoimmune origin. Exposure to an MMTV-like agent is improbable because (a) low frequencies of reactions to p27 and gp52 (about 2% for p27 and 3% for gp52 of all cancer patients sera tested and (b) we did not find any sera containing both types of antibodies i.e. to p27 and gp52. The autoimmune response might be directed against the product of activated endogenous retrovirus related to MMTV or against some cellular protein that shares partial sequence homology with viral proteins. The latter situation has been described for autoimmune reactions of sera from patients with mixed connective tissue disease recognising the 70 kDa protein of $U_1$snRNP immunologically cross-reactive with p30 MuLV (Query & Keene, 1987). The conclusions can be summarised as follows: (1) specific antibodies recognising MMTV proteins sporadically appear in human sera. (2) Most frequent reactivities are to transframe protein p30 and ribonucleoprotein p14. These reactions occur in sera of cancer patients and healthy controls. (3) Specific reactions to major core protein p27 and envelope glycoprotein gp52 were observed predominantly in sera of cancer patients but their prevalence is low. (4) Reactions with 42 kDa cellular contaminant of the virus occur more frequently than specific reactions to MMTV proteins and they might be responsible for false positivities in ELISA and CIF assays. (5) No direct correlation between the incidence of malignant disease and the presence of specific antibodies to MMTV proteins can be found due to their low incidence. Nevertheless, their sporadic occurrence may help further search for antigens circumstantially expressed during carcinogenesis.

We would like to express our gratitude to Dr B. Westley from the University of Newcastle Upon Tyne for his kind review of the manuscript. The excellent technical assistance of Miss M. Janoťáková, Miss A. Chrkavá and Mrs H. Görnerová is greatly appreciated.

### References

AL-SUMIDAIE, A.M., LEINSTER, S.J., HART, C.A., GREEN, C.D. & McCARTHY, K. (1988). Particles with properties of retroviruses in monocytes from patients with breast cancer. *Lancet*, **i**, 5.

BARBACID, M., BOLOGNESI, D. & AARONSON, S.A. (1980). Humans have antibodies capable of recognizing oncoviral glycoproteins: Demonstration that these antibodies are formed in response to cellular modification of glycoproteins rather than as a sequence of exposure to virus. *Proc. Natl Acad. Sci. USA*, **77**, 1617.

BITTNER, M., KUPFERER, P. & MORRIS, C.F. (1980). Electrophoretic transfer of proteins and nucleic acids from slab gels to diazobenzyloxymethyl cellulose or nitrocellulose sheets. *Anal. Biochem.*, **102**, 459.

CALLAHAN, R., DROHAN, W., TRONICK, S. & SCHLOM, J. (1982). Detection and cloning of human DNA sequences related to the mouse mammary tumor virus genome. *Proc. Natl Acad. Sci. USA*, **79**, 5503.

DAY, N.K., WITKIN, S.S., SARKAR, N.H. & 6 others (1981). Antibodies reactive with murine mammary tumor virus in sera of patients with breast cancer: geographic and family studies. *Proc. Natl Acad. Sci. USA*, **78**, 2483.

DEEN, K.C. & SWEET, R.W. (1986). Murine mammary tumor virus pol-related sequences in human DNA: characterization and sequence comparison with the complete murine mammary tumor virus pol gene. *J. Virol.*, **57**, 422.

DION, A.C., GIRARDI, A.J., WILLIAMS, C.C., POMENTI, A.A. & REDFIELD, E.S. (1987). Responses of serum from breast cancer patients to murine mammary tumor virus: fact or artifact? *J. Natl Cancer Inst.*, **79**, 207.

FRANKLIN, G.C., CHRETIEN, S., HANSON, I.M., ROCHEFORD, H., MAY, F.E.B. & WESTLEY, B.R. (1988). Expression of human sequences related to those of mouse mammary tumor virus. *J. Virol.*, **62**, 1203.

GREENWOOD, F.C., HUNTER, W.M. & GLOVER, J.S. (1963). The preparation of $^{131}$I-labelled human growth hormone of high specific radioactivity. *Biochem. J.*, **89**, 114.

HIZI, A., HENDERSON, L.E., COPELAND, T.D., SOWDER, R.C., HIXON, C.V. & OROSZLAN, S. (1987). Characterization of mouse mammary tumor virus gag-pro gene products and the ribosomal frameshift site by protein sequencing. *Proc. Natl Acad. Sci. USA*, **84**, 7041.

HOLDER, W.D. JR & WELLS, S.A. JR (1983). Antibody reacting with the murine mammary tumor virus in the serum of patients with breast carcinoma: a possible serological detection method for breast carcinoma. *Cancer Res.*, **43**, 239.

KEYDAR, T., OHNO, T., NAYAK, R. & 6 others (1984). Properties of retroviruses-like particles produced by a human breast carcinoma cell line: immunological relationship with mouse mammary tumor virus proteins. *Proc. Natl Acad. Sci. USA*, **81**, 4188.

KOVARIK, A., HLUBINOVA, K., VRBENSKA, A. & PRACHAR, J. (1987). An improved colloidal silver staining method of protein blots on nitrocellulose membranes. *Folia Biol (Prague)*, **33**, 253.

LAEMMLI, U.K. (1970). Cleavage of structural proteins during assembly of the head of bacteriophage T4. *Nature*, **227**, 680.

ONO, M., KAWAKAMI, M., & USHIKUBU, H. (1987). Stimulation of expression of the human endogenous retrovirus genome by female steroid hormones in human breast cancer line T47D. *J. Virol.*, **60**, 589.

ONO, M., YASUNAGA, T., MIYATA, T. & USHIKUBU, H. (1986). Nucleotide sequence of human endogenous retroviral genome related to the mouse mammary tumor virus genome. *J. Virol.*, **60**, 589.

QUERY, C.C. & KEENE, J.D. (1987). A human autoimmune protein associated with U1 RNA contains a region homology that is cross-reactive with retroviral $p30^{gag}$ antigen. *Cell*, **51**, 211.

SALMONS, B. & GÜNZBURG, W.H. (1987). Current perspectives in the biology of mouse mammary tumor virus. *Virus Res.*, **8**, 81.

SHOENFELD, Y., HIZI, A., TAL, R. & 7 others (1987). Human monoclonal antibodies derived from lymph nodes of a patient with breast carcinoma react with MuMTV polypeptides. *Cancer*, **59**, 43.

SNYDER, H.W. JR & FLEISSNER, E. (1980). Specificity of a human antibodies to oncovirus glycoproteins: recognition of antigen by natural antibodies directed against carbohydrate structures. *Proc. Natl Acad. Sci. USA*, **77**, 1622.

SYU, W.J. & KAHAN, L. (1987). Use of protein-stained immunoblots for unequivocal identification of antibody specificities. *J. Immunol. Methods*, **103**, 247.

TOMANA, M., KAJDOS, A., NIEDERMEIER, W., DURKIN, W. & MASTECKY, J. (1981). Antibodies to mouse mammary tumor virus-related antigen in sera of patients with breast carcinoma. *Cancer*, **47**, 2696.

WITKIN, S.S., SARKAR, N.H., KINNE, D.W., GOOD, R.A. & DAY, N.K. (1980). Antibodies reactive with mouse mammary tumor virus in sera of patients with breast cancer. *Int. J. Cancer*, **25**, 721.

ZOTTER, S., GROSSMANN, H., FRANCOIS, C. & 4 others (1983). Among the human antibodies reacting with intracytoplasmatic A particles of mouse mammary tumor virus, some react with p14, the nucleic acid binding protein, and others with p28, the main core protein. *Int. J. Cancer*, **32**, 27.